# Histopathological Diagnostic Discrepancies in Soft Tissue Tumours Referred to a Specialist Centre

**DOI:** 10.1155/2009/741975

**Published:** 2009-05-27

**Authors:** Khin Thway, Cyril Fisher

**Affiliations:** Department of Histopathology, Royal Marsden Hospital, London SW3 6JJ, UK

## Abstract

*Aims*. A study was performed to determine areas of diagnostic discrepancy in the reporting of cases of soft tissue tumours referred to a specialist sarcoma unit. This was to pinpoint common discrepancies and to determine their causes. *Methods and Results*. We compared the sarcoma unit's histopathology reports with referring reports on 349 specimens from 277 patients with suspected or proven soft tissue tumours in a one-year period. *Conclusions*. Diagnostic agreement was found in 256 of 349 cases (73.4%), with minor diagnostic discrepancy in 55 cases (15.7%) and major discrepancy in 38 cases (10.9%). Benign/malignant discordances accounted for only 5% of all discrepancies (5 cases). The most common discrepancies occurred in tumour classification, including diagnosis of gastrointestinal stromal tumour and leiomyosarcoma and the subtyping of spindle cell sarcomas, as well as in tumour grading that could conceivably lead to changes in clinical management. Major diagnostic discrepancies leading to management change occurred in a relatively select range of tumour groups, and almost all discrepancies occurred due to differences in tumour interpretation between general or nonsoft tissue pathologists, and pathologists at the specialist unit. The findings support guidelines by the National Institute for Health and Clinical Excellence that diagnostic review of soft tissue tumours should be performed by specialist soft tissue pathologists.

## 1. Introduction

Soft tissue tumours are diagnostically challenging, with histopathological criteria that are constantly evolving, particularly concerning ancillary investigations such as immunohistochemistry and molecular genetics. Much of the diagnostic challenge is due to their rarity: the United Kingdom has an annual incidence of about 2.5 per 100 000 population, and the average general pathologist might encounter only one or two sarcomas throughout the course of a year. The Royal Marsden Hospital (RMH) is a tertiary cancer treatment centre which receives referrals regionally, nationally, and internationally. The Sarcoma Unit at RMH takes approximately 1200 new histopathology accessions per year (excluding second opinion cases), of which about 350 are referral cases. In line with hospital policy, all new patients with suspected or proven soft tissue tumours have their histology reviewed by a specialist soft tissue pathologist. By comparing Sarcoma Unit and referring reports, diagnostic discrepancies were established, their reasons evaluated, and diagnostic areas especially prone to problems described. Previous studies have focussed on differences between second opinion and referring reports, including sarcoma second opinion cases [1]. Second opinion cases are distinct from referrals, as they are mostly sought by pathologists, with smaller numbers requested by clinicians: these cases are usually rarer and more diagnostically challenging to pathologists without special interest in soft tissue. Referral cases are mainly those diagnosed by the local pathologist, on which diagnosis the patient is then referred to a specialist centre. These cases, in the main, represent less diagnostic challenge than those sent for second opinion. Histopathological peer reviews for sarcoma diagnoses have been published by Presant et al. in the South East of the United States [2], by Alvegard and Berg for the Scandinavian Sarcoma Group [3], and by Harris et al. in the North West of England [4], but no recent similar audits for soft tissue tumours exist. A contemporary study is pertinent, since there has been considerable evolution in the methods and criteria used for diagnosis of soft tissue tumours.

## 2. Materials and Methods

A retrospective audit was performed for patients referred to a specialist soft tissue sarcoma unit with soft tissue tumours over a one-year period. The record files within the Department of Histopathology at the Royal Marsden Hospital were examined for a period of 12 months from the 1st of January to the 31st of December 2005. Patients were either surgical or oncological referrals. Referrals to the surgical unit were usually patients with a new histological diagnosis after biopsy, or those with recurrent lesions referred for further surgery. Patients were referred to the medical or clinical oncology units for planning of (neo)adjuvant treatment. All second opinion cases (including those sent for pathological opinion, but where the patient was not referred) were excluded, as were cases without referring reports. All cases included in the study were reviewed during the time period assessed by one of the authors (C.F.), and any cases requiring subsequent review were assessed by both authors. Each referring report was compared with the subsequent Sarcoma Unit report for differences in diagnosis and grading. Grading was assigned according to the system by the French Federation of Cancer Centres Sarcoma Group (FNCLCC) [5, 6]. Grading categories were defined as (1) not applicable, (2) not done, (3) no difference in grade, (4) difference by one grade, and (5) difference by two grades. For gastrointestinal stromal tumour (GIST), assessment of potential biological behaviour into low, intermediate, and high risk was also compared as for grading. Tumours for which the referring pathologist had identified tumour type, performed a mitotic count and reported on the absence or amount of necrosis but had not given a numerical grade, were retrospectively graded on review and recorded as “graded.” Tumours for which the mitotic count had not been performed, or presence or absence of necrosis not indicated or where neither had been done, were not retrospectively graded. Tumours which the referring pathologist had assigned as low, intermediate, and high grade were interpreted as grade 1, 2, and 3 respectively. Grading was deemed as “not applicable” in (1) certain sarcomas considered routinely to display aggressive or “high-grade” behaviour, (2) metastatic tumours, (3) tumours not formally graded, such as dermatofibrosarcoma protuberans, (4) benign lesions, (5) if there was a difference in diagnosis between the referring and specialist unit report, making grading noncomparable, or (6) there was insufficient material for grading.

Major discrepancies were defined as those that could lead to significant change in clinical management, with ensuing under- or overtreatment, and were divided into six groups: (1) malignant → malignant (resulting in significant management change), (2) malignant → benign, (3) benign → malignant, (4) mesenchymal → nonmesenchymal, (5) other (e.g., benign → benign, but resulting in significant management change), and (6) major grading discrepancies, comprising tumours in which there was any interchange of grade between grades 2-3 and grade 1 (as this could lead to management change). 

Minor discrepancies were divided into those of diagnosis, classification, or grading, but they were those in which the discrepancy was not thought to provoke significant management change. Minor changes in which the discrepancy was purely semantic, or in Sarcoma Unit reports in which subcategorisation was chiefly for special or academic interest (e.g., the addition of a finding of myofibroblastic differentiation within pleomorphic sarcoma), were disregarded. The reasons for discrepancy were analysed, by further assessing reports and reviewing slides where appropriate or possible, to look for sources of error such as interpretation of morphology or immunohistochemistry. 

## 3. Results

In the 12-month period studied, there were 277 referral patients with suitable material for auditing, with a total of 349 specimens. Some patients had multiple specimens assessed in this time period. These included patients who had repeat core biopsies, who had initial core biopsies proceeding later to incisional/open biopsies, or who had core biopsies with subsequent resection specimens. Of the specimens, 199 were oncological referrals, and 150 were surgical. 203 specimens were from district general hospitals, 120 from teaching hospitals, and 26 from overseas hospitals. 256 of 349 cases (73.4%) showed diagnostic agreement between referring and Sarcoma Unit reports. 93 cases (26.6%) showed discrepancy between referring and Sarcoma Unit reports. There was no correlation between incidence of discrepancy and type of referring institution. 47 cases were oncological referrals, and 46 were surgical. 38 of the 93 discrepant cases were major discrepancies (10.9% of total or 40.9% of discrepancies; see Table 1), and 55 were minor (15.7% of total, or 59.1% of discrepancies; see Table 2). 

Of the 38 major discrepancies, 17 cases were diagnosed on resection material, 11 on excisional or incisional biopsies, and ten on needle core biopsies. Of the six groups of major discrepancy, the largest was malignant → malignant, accounting for 15 cases (39.5% of discrepancies in this group). Within this group, the most common discrepant diagnosis was for GIST (seven cases; 18.4% of major diagnostic discrepancies). The next largest group was grading discrepancies (nine cases), followed by mesenchymal → nonmesenchymal discrepancy (seven cases), benign → malignant (three cases), and malignant → benign and other (each two cases).

Of the 55 minor discrepancies, 28 were resections, 15 were excision biopsies, and 12 needle core biopsies. Of these, 29 (52.7%) were diagnostic differences, 18 (32.7%) were classification differences, and eight (14.5%) were differences in grading. Some patterns emerged in the types of discrepancies encountered, and particular areas of emphasis are discussed below.

## 4. GIST

Diagnoses were changed to leiomyosarcoma (three cases), dedifferentiated liposarcoma (three cases), and Kaposi sarcoma (one case). The main reason for diagnostic discrepancy was misinterpretation of immunohistochemistry for CD117 (two cases each of leiomyosarcoma and dedifferentiated liposarcoma, and the case of Kaposi sarcoma), while two cases were due to morphological misinterpretation. In two cases of leiomyosarcoma, smooth muscle markers were positive, but faint diffuse nonspecific staining for CD117 had been interpreted as positive. In two cases of dedifferentiated liposarcoma, similar faint CD117 staining was interpreted as positive, in the absence of positivity for other markers. In the other case, CD117 was correctly interpreted as negative, but the diagnosis was based on morphology. In the case of Kaposi sarcoma, diffuse CD34 positivity (in addition to focal CD117 positivity) led to misinterpretation; subsequent positivity for antibody to Human Herpesvirus 8 (HHV-8) led to reclassification of the tumour at Sarcoma Unit. In addition, three cases were changed to GIST following review (two leiomyosarcoma, one cellular neurothekeoma). Although morphology of all cases was adequately described, the two cases diagnosed as leiomyosarcomas, from the stomach and peritoneum, respectively, showed focal SMA positivity, while CD117 had not been performed. The cellular neurothekeoma had been diagnosed on morphology alone, without ancillary immunohistochemistry. Morphology was not considered consistent with this on review.

## 5. Smooth Muscle Tumours

Misinterpretation of cases as leiomyosarcoma accounted for 4/38 (10.5%) of major discrepancies, with two cases reclassified as GIST following review, and one case each reclassified as alveolar rhabdomyosarcoma and malignant solitary fibrous tumour. The cases reclassified as GIST showed characteristic morphology and diffuse CD117 positivity on immunohistochemistry at Sarcoma Unit, as described above. Alveolar rhabdomyosarcoma had been diagnosed as leiomyosarcoma on the strength of diffuse desmin positivity, although smooth muscle marker immunohistochemistry had not been performed, and the morphology was inconsistent. The case reclassified as solitary fibrous tumour originated from the broad ligament and had been diagnosed on morphology alone. As to cases initially reported as nonmalignant smooth muscle tumours, one case diagnosed as atypical leiomyoma was reclassified as low-grade endometrial stromal sarcoma (ESS). This showed focal SMA positivity, but on review, typical ESS morphology was noted, and diffuse CD10 positivity. One case reported as leiomyoma on core biopsy was reclassified as leiomyosarcoma on review: the occasional atypia reported by the referring institution was interpreted as diffuse atypia at Sarcoma Unit.

## 6. Liposarcoma

Two cases were referred with diagnoses of well-differentiated liposarcoma, changed to dedifferentiated liposarcoma following review. Of these, one showed several foci of low-grade dedifferentiation. The other, originally diagnosed as the sclerosing form of well-differentiated liposarcoma, showed, on review, several low-power fields with atypical, moderately cellular tumour consistent with dedifferentiation. 

## 7. Myofibroblastic Lesions

Only two myofibroblastic lesions were present within the major discrepancy group, and neither of these was mistaken for frank sarcoma. Both (one case, from the abdominal cavity, originally diagnosed as inflammatory myofibroblastic tumour, and one case, from the calf, diagnosed as nodular fasciitis) were interpreted as fibromatosis following review. Both cases showed the typical sweeping fascicular morphology of fibromatosis, and error was probably due to morphological unfamiliarity.

## 8. Nonmesenchymal Lesions

7/38 (18.4%) of major discrepancies were due to cases referred as sarcoma or possible sarcoma and reclassified at Sarcoma Unit as nonmesenchymal lesions. The cases were almost all poorly differentiated neoplasms, with final diagnoses of one each of carcinoma, metaplastic carcinoma, carcinoma/germ cell tumour, germ cell tumour, metastatic seminoma, metastatic melanoma, and diffuse large B-cell lymphoma.

## 9. Grading

One tumour was downgraded by two grades. Five tumours were upgraded from grade 1 to 2, and three tumours were downgraded from grade 2 to 1. All of these tumours were leiomyosarcomas.

## 10. Classification

Seven of 18 classification discrepancies involved smooth muscle tumours. Four of these were malignant lesions and comprised one case of pleomorphic leiomyosarcoma reclassified as leiomyosarcoma (NOS), one case of smooth muscle tumour of low malignant potential reclassified as leiomyosarcoma, grade 2, one case of cutaneous leiomyosarcoma reclassified as leiomyosarcoma (noncutaneous), and one case of leiomyosarcoma reclassified as cutaneous leiomyosarcoma.

## 11. Discussion

The total discrepancy rate in this study was 26.6%. In comparison, the Southeastern Cancer Study Group reported a 28% disagreement rate between primary institutional diagnosis and reviewer diagnosis [2]. The Scandinavian Sarcoma Group reported that 25% of sarcomas they reviewed were reclassified, and that in 40% the grade was changed [3]. The North West England peer review in 1991 showed a discrepancy rate of approximately 35% [4] (disagreement in subtype in 17% and change in diagnosis to nonsarcomatous tumours in 18%), with an agreement rate of sarcoma subtype of 53%, and the remaining cases accounting for tumours where subtype could not be further specified, where classification was only possible as “malignant tumour NOS,” or where diagnosis could not be given. The 2001 audit of soft tissue second opinion cases by Arbiser et al. showed major discrepancy in 25% of cases and minor discrepancy in 7% [1]. 

Interestingly, benign/malignant discrepancies only accounted for a small number of total discrepancies in this study. Most major discrepancies were due to differences of opinion in tumour categorisation. This implies that major discrepancies are due to unfamiliarity of pathologists with certain tumours, which may be reflected in selection of inappropriate immunopanels, rather than, more crudely, missing frankly malignant or frankly benign morphological features. As previously mentioned, certain patterns emerge, with some neoplasms more prone to misdiagnosis than others. 

From the authors' experience (unpublished observations), misinterpretation of lesions as GIST can occur relatively frequently, with both benign tumours, such as fibromatosis, and malignant tumours being erroneously diagnosed. Conversely, GIST is also often mistaken for other tumours, particularly because of the range of immunohistochemical markers it can express, and the frequent presence of diffuse positivity for CD34 and/or h-caldesmon might sway the pathologist into presuming vascular, fibroblastic or smooth muscle differentiation. Misinterpretation of immunostaining for CD117 however remains the main reason for diagnostic error, both with other CD117-positive tumours such as Kaposi sarcoma [7] and with nonspecific weak background staining for this marker (and occasional cytoplasmic positivity in myofibroblastic lesions) [8] which is often mistaken for diffuse positivity. Use of newer reagents, such as DOG1 [9], and of mutational analysis, might be expected to reduce this discrepancy. Positivity in tumours with high numbers of mast cells may rarely cause confusion. Owing to the common and effective use of targeted therapy with imatinib, GIST is high on the list of differential diagnoses for abdominal and retroperitoneal tumours for both clinicians and pathologists, whereas other tumours in these sites, such as dedifferentiated liposarcoma, might be less anticipated by nonsoft tissue physicians. Reasons for confusing GIST with leiomyosarcoma are apparent: the morphological appearances can be similar, as both are spindle cell lesions with fascicular architectures and with cells showing paranuclear vacuolation. There is also overlap in smooth muscle marker expression: in addition to SMA positivity, present focally in up to 47% of GISTs, diffuse, strong positivity for h-caldesmon is seen in many. 

Although well-differentiated and dedifferentiated liposarcomas are opposite ends of one disease spectrum, they may be difficult to distinguish, and low-grade dedifferentiation, which is thought to exhibit similar biologic behaviour to that of its high-grade counterpart, might on occasion be sparsely cellular [10]. Discrepancies in categorisation between well-differentiated liposarcoma and dedifferentiated liposarcoma, even if dedifferentiation is low grade, were placed in the “major discrepancy” category, as sclerosing well-differentiated liposarcoma is a grade 1 tumour with a low recurrence risk, whereas dedifferentiated liposarcoma has a much greater risk of recurrence as well as metastasis. Three cases of liposarcoma were previously reported as GISTs, as described above. One case diagnosed as lipoma was changed to well-differentiated liposarcoma following Sarcoma Unit review. This was situated deeply in the neck: features of spindle cell lipoma were not noted, and although in a relatively unusual site, the lesion showed the characteristic septate morphology with enlarged, hyperchromatic spindle cells typical of well-differentiated liposarcoma. One case diagnosed as well-differentiated liposarcoma was considered lipoma with fat necrosis on review. Fat necrosis can often confuse, and vacuolations within macrophages are frequently mistaken for lipoblasts. 

Germ cell tumours accounted for three of seven nonmesenchymal lesions. They were all in abdominopelvic/retroperitoneal locations, emphasising that metastatic germ cell tumour should be considered in the differential diagnosis of poorly differentiated tumours arising in these sites. In diffuse large B-cell lymphoma, the presence of dispersed atypical cells, particularly within sclerotic stroma that might itself contain enlarged, reactively atypical fibroblastic/myofibroblastic spindle cells, often showing strong and diffuse expression of SMA, can lead to confusion with spindle cell sarcoma. Although easily distinguished with pan-B-cell markers, an index of suspicion for lymphoma is required. 

Of the 29 minor diagnostic discrepancies (Table 2(a)), pleomorphic sarcoma (so-called malignant fibrous histiocytoma) accounted for the diagnosis that tumours were most frequently changed to. Four cases were originally diagnosed as leiomyosarcoma. On review, all showed focal SMA positivity, often in subplasmalemmal distributions, and were negative for desmin and h-caldesmon, consistent with myofibroblastic differentiation. Typical leiomyosarcomatous morphology (intersecting fascicles of spindle cells, possibly with blunt-ended nuclei and paranuclear vacuolations) was absent in each case. Pleomorphic sarcoma was also initially diagnosed as rhabdomyosarcoma (two cases), extraskeletal osteosarcoma, and mesenchymal chondrosarcoma (one case each). 

Leiomyosarcoma/pleomorphic leiomyosarcoma was the diagnosis most frequently changed following Sarcoma Unit review (nine cases). The differences probably occurred because the tertiary centre pathologists had higher thresholds and stricter criteria for diagnosing leiomyosarcoma, including strong and diffuse smooth muscle marker immunohistochemistry, and categorisation as pleomorphic leiomyosarcoma only when conventional leiomyosarcoma was present in other areas of the tumour. Interpretation of tumoural osteoid is another difficult area, as is lipoblast identification. Vacuolated fibroblasts present in myxofibrosarcoma, and foamy macrophages in fat necrosis are often interpreted as lipoblasts, although both lack the nuclear indentation of the latter. Most of these diagnostic distinctions are, arguably, relatively academic: the majority of cases remain high-grade sarcomas regardless of subcategorisation, and although there might be minor prognostic differences based on subsequent diagnosis, treatment would not differ significantly in most cases. 

44 of 68 gradable malignant soft tissue tumours with sufficient material were given a Trojani grade by the referring pathologists. Grading was not obtainable in 13 additional cases, where there was insufficient material, for example, a very small amount of tissue in a needle core biopsy or tumour accounting for only a small portion of material in the core. 25% of sarcomas were regraded, including five from grade 1 to grades 2/3 and four from grades 2/3 to grade 1. This is of clinical significance, as grade 1 sarcomas are in general treated by excision, whereas grade 2 tumours are treated more aggressively, including with adjuvant radiotherapy. 

The availability of ancillary tests was not, as might be expected, a cause of significant discrepancies. Virtually all common immunohistochemical markers for soft tissue tumour diagnosis are routinely available within the laboratories of district general and teaching hospitals, and no diagnostic discrepancy was knowingly noted to occur due to a department lacking a particular antibody. Similarly, no discrepancy occurred because of a subsequent positive result on molecular genetic analysis at RMH. In both major and minor discrepancy groups, the majority of specimens were excisions, that is, there was sufficient lesional material for diagnosis. No difference in rate was noted between referrals from district or teaching hospitals. 

Almost all discrepancies therefore occurred due to differences in interpretation, either of morphology or immunohistochemistry. Part of the challenge in soft tissue diagnosis is maintaining a suitable rate of exposure to cases, and this of course is difficult for the majority of pathologists who do not work at tertiary soft tissue or bone centres. Previous studies in the North West of England hypothesised the incidence of soft tissue sarcoma there as 18 per 10^6^ person years, meaning approximately 70 new cases per year in this region [11, 12]. Histopathologists specialising in just one or a few fields are more likely to know and be able to categorise these in detail than the generalist. A pathologist seeing a high volume of similar cases also, through experience, might routinely perform CD117 on all abdominal or retroperitoneal spindle cell lesions. Similarly, when diagnosing pleomorphic or spindle cell sarcoma without specific differentiation in these locations, the soft tissue pathologist, knowing that many such tumours here represent dedifferentiated liposarcoma, would look for an adjacent well-differentiated component. Although not significantly noted in this study, other areas in which there might be difficulty include unusual (e.g., myxoid) variants of typical lesions. A further issue is that a pathologist working in a referral centre already has a “referral bias” or index of suspicion, that is, a case coming in is already flagged as a potential sarcoma, in contrast to a general pathologist faced with one case within a general surgical pathology workload. 

The Guidelines for Cancer Services issued by the National Institute for Health and Clinical Excellence (NICE) in 2006 indicate that all soft tissue sarcomas should be either first reported or reviewed by a specialist soft tissue sarcoma pathologist, who regularly reports soft tissue tumours as a significant component of their workload, takes part in external quality assessment (EQA), and is a member of a properly constituted sarcoma multidisciplinary team (MDT) [13]. All patients with soft tissue tumours assessed in a diagnostic clinic should have their pathology reported by either a specialist soft tissue pathologist or a pathologist nominated by the sarcoma MDT as part of the local diagnostic referral pathway, who has formal links to a specialist soft tissue pathologist. Commissioners should fund a formal system for second opinions and review of difficult cases, including the use of molecular and cytogenetic facilities.

This audit emphasises that specific groups of interpretative discrepancies in soft tissue tumours exist and suggests that soft tissue tumours should be reported by or referred to specialist soft tissue pathologists, as stated in the 2006 NICE guidelines. The study relates to a period before these guidelines were in place.

## 12. Conclusions

The purpose of this audit was to pinpoint common discrepancies in soft tissue tumour diagnosis and to find causes for these. Certain tumours were more liable to misinterpretation and included GIST, leiomyosarcoma, and pleomorphic sarcoma. There was no significant correlation between type of referring institution and discrepancy rate, and diagnostic disagreements were as frequent in resection material as needle core biopsy. Almost all discrepancies occurred due to interpretational differences, either of tumour morphology or of immunophenotype. There was no case in which the discrepancy was conclusively due to lack of available resources at the referring institution, such as immunohistochemistry or other ancillary investigations, and this suggests that reasons for disagreement are due to unfamiliarity in dealing with rare cases or lack of awareness of certain site-specific tumours. While the majority of tumours are accurately diagnosed by referring pathologists, the discrepancy rate of almost 27% highlights that, as recommended by NICE guidelines, it remains important to review the histopathology of all patients referred to specialist soft tissue tumour centres.

## Figures and Tables

**(a) Malignant → Malignant:** 

**Previous Diagnosis**	**Final Diagnosis**	**Number of cases**
GIST	Leiomyosarcoma	3
	Liposarcoma, dedifferentiated	2
	Liposarcoma, well differentiated	1
	Kaposi sarcoma	1
Leiomyosarcoma	GIST	2
	SFT, malignant	1
	Alveolar RMS	1
Atypical leiomyoma	Endometrial stroma sarcoma	1
Rhabdomyosarcoma	Ewing sarcoma	1
Liposarcoma, WD	Liposarcoma, dedifferentiated	2

**(b) Grading:** 

**Previous Grade**	**Final Grade**	**Number of cases**
Leiomyosarcoma, grade 3	Grade 1	1
Leiomyosarcoma, grade 2	Grade 1	3
Leiomyosarcoma, grade 1	Grade 2	5

**(c) Mesenchymal → Nonmesenchymal:** 

**Previous Diagnosis**	**Final Diagnosis**	**Number of cases**
DSRCT	Carcinoma/GCT	1
Granuloma ? Sarcoma	Seminoma, metastatic	1
? Sarcoma	GCT	1
? Sarcoma ? Melanoma	Anaplastic carcinoma	1
? Sarcoma	DLBCL	1
Chondrosarcoma	Metaplastic carcinoma	1
MPNST	Melanoma, metastatic	1

**(d) Benign → Malignant:** 

**Previous Diagnosis**	**Final Diagnosis**	**Number of cases**
Cellular neurothekeoma	GIST	1
Leiomyoma	Leiomyosarcoma	1
Lipoma	Liposarcoma, WD	1

**(e) Malignant → Benign:** 

**Previous Diagnosis**	**Final Diagnosis**	**Number of cases**
Low-grade fibromyxoid sarcoma	Superficial angiomyxoma	1
Liposarcoma, WD	Lipoma	1

**(f) Other:** 

**Previous Diagnosis**	**Final Diagnosis**	**Number of cases**
IMT	Fibromatosis	1
Nodular fasciitis	Fibromatosis	1

**(a) Diagnostic discrepancies:** 

**Previous Diagnosis**	**Final Diagnosis**	**Number of cases**
Leiomyosarcoma	Pleomorphic sarcoma	4
	Spindle cell sarcoma, myofibroblastic differentiation	2
	Myofibrosarcoma	1
	Myxofibrosarcoma	1
Leiomyosarcoma, pleomorphic	Spindle cell sarcoma	1
Rhabdomyosarcoma	Pleomorphic sarcoma, myogenic differentiation	1
	Pleomorphic sarcoma, smooth muscle differentiation	1
Liposarcoma, pleomorphic	Myxofibrosarcoma	2
	Myxoid liposarcoma	1
Liposarcoma, dedifferentiated	Spindle cell rhabdomyosarcoma	1
Synovial sarcoma	SFT, malignant	1
Extraskeletal osteosarcoma	Pleomorphic sarcoma	1
Chondrosarcoma	Extraskeletal osteosarcoma with focal chondrosarcomatous areas	1
Mesenchymal chondrosarcoma	Pleomorphic sarcoma	1
Epithelioid haemangioendothelioma	Epithelioid haemangioma	1
Sarcoma NOS	SFT	1
Spindle cell sarcoma	Leiomyosarcoma	1
Pleomorphic sarcoma	Myxoinflammatory fibroblastic sarcoma	1
Spindle cell proliferation	Fibromatosis	2
	Fibrosarcoma, grade 1	1
	IMT	1
Spindle cell proliferation, atypical	Leiomyosarcoma, grade 1	1
	Spindle cell sarcoma NOS	1

**(b) Classification discrepancies:** 

**Previous Diagnosis**	**Final Diagnosis**	**Number of cases**
Smooth muscle tumour, low malignant potential	Leiomyosarcoma, grade 2	1
Leiomyosarcoma	Cutaneous leiomyosarcoma	1
Leiomyosarcoma, cutaneous	Leiomyosarcoma (non-cutaneous)	1
Pleomorphic leiomyosarcoma	Leiomyosarcoma	1
Uterine fibroid	Smooth muscle tumour of uncertain malignant potential	1
Leiomyosarcoma, grade 1	Atypical leiomyoma	1
	Smooth muscle tumour of uncertain malignant potential	1
Rhabdomyosarcoma	Pleomorphic sarcoma with myogenic differentiation	1
Pleomorphic rhabdomyosarcoma	Embryonal rhabdomyosarcoma, anaplastic variant	1
	Alveolar rhabdomyosarcoma	1
	Pleomorphic sarcoma	1
Liposarcoma	Myxoid-round cell liposarcoma, grade 2	1
PNET	Primitive malignant tumour with rhabdomyosarcomatous and neural differentiation	1
Endometrial stromal sarcoma, high grade	Endometrial stromal sarcoma, low grade	1
Pleomorphic sarcoma, rhabdomyosarcomatous differentiation	Pleomorphic sarcoma, myofibroblastic differentiation	1
? Sarcoma ? carcinoma	Pleomorphic sarcoma	1
	Synovial sarcoma	1
Pleomorphic sarcoma	Myxofibrosarcoma	1

**(c) Grading discrepancies:** 

**Previous Grade**	**Final Grade**	**Number of cases**
Leiomyosarcoma, grade 2	Grade 3	2
Pleomorphic liposarcoma, grade 3	Grade 2	3
Pleomorphic sarcoma, grade 3	Grade 2	2
GIST, high risk	Intermediate risk	1

## Abbreviations

DLBCL: Diffuse large B-cell lymphoma
DSRCT: Desmoplastic small round cell tumour
GIST: Gastrointestinal stromal tumour
IMT: Inflammatory myofibroblastic tumour
MPNST: Malignant peripheral nerve sheath tumour
NOS: Not otherwise specified
PNET: Primitive neuroectodermal tumour
RMS: Rhabdomyosarcoma
SFT: Solitary fibrous tumour
